# Supplementary material for: Cuproptosis-related gene signatures define the immune microenvironment in diabetic nephropathy
Source: PLoS One. 2025 Jun 3;20(6):e0321636. doi: 10.1371/journal.pone.0321636 (PMC12133194; doi:10.1371/journal.pone.0321636)
Supplement: Attachment — Submitted filename: Response to reviewer.doc (XLSX) [file pone.0321636.s009.doc]

PONE-D-24-19973

Cuproptosis gene characterizes the immune microenvironment of diabetic nephropathy.

PLOS ONE

Dear Dr. lu,

Thank you for submitting your manuscript to PLOS ONE. After careful consideration, we feel that it has merit but does not fully meet PLOS ONE’s publication criteria as it currently stands. Therefore, we invite you to submit a revised version of the manuscript that addresses the points raised during the review process.

Comments from PLOS Editorial Office: We note that one or more reviewers has recommended that you cite specific previously published works. As always, we recommend that you please review and evaluate the requested works to determine whether they are relevant and should be cited. It is not a requirement to cite these works. We appreciate your attention to this request.

Please submit your revised manuscript by Aug 05 2024 11:59PM. If you will need more time than this to complete your revisions, please reply to this message or contact the journal office at <a href="mailto:plosone@plos.org">plosone@plos.org. When you're ready to submit your revision, log on to https://www.editorialmanager.com/pone/ and select the 'Submissions Needing Revision' folder to locate your manuscript file.

Please include the following items when submitting your revised manuscript:

A rebuttal letter that responds to each point raised by the academic editor and reviewer(s). You should upload this letter as a separate file labeled 'Response to Reviewers'.

A marked-up copy of your manuscript that highlights changes made to the original version. You should upload this as a separate file labeled 'Revised Manuscript with Track Changes'.

An unmarked version of your revised paper without tracked changes. You should upload this as a separate file labeled 'Manuscript'.

If you would like to make changes to your financial disclosure, please include your updated statement in your cover letter. Guidelines for resubmitting your figure files are available below the reviewer comments at the end of this letter.

If applicable, we recommend that you deposit your laboratory protocols in protocols.io to enhance the reproducibility of your results. Protocols.io assigns your protocol its own identifier (DOI) so that it can be cited independently in the future. For instructions see: https://journals.plos.org/plosone/s/submission-guidelines#loc-laboratory-protocols. Additionally, PLOS ONE offers an option for publishing peer-reviewed Lab Protocol articles, which describe protocols hosted on protocols.io. Read more information on sharing protocols at https://plos.org/protocols?utm_medium=editorial-email&utm_source=authorletters&utm_campaign=protocols.

We look forward to receiving your revised manuscript.

Kind regards,

Qi Zhao

Academic Editor

PLOS ONE

Journal Requirements:

When submitting your revision, we need you to address these additional requirements.

1. Please ensure that your manuscript meets PLOS ONE's style requirements, including those for file naming. The PLOS ONE style templates can be found at

https://journals.plos.org/plosone/s/file?id=wjVg/PLOSOne_formatting_sample_main_body.pdf and

https://journals.plos.org/plosone/s/file?id=ba62/PLOSOne_formatting_sample_title_authors_affiliations.pdf

2. Please note that funding information should not appear in any section or other areas of your manuscript. We will only publish funding information present in the Funding Statement section of the online submission form. Please remove any funding-related text from the manuscript.

3. Thank you for stating the following financial disclosure:

"This work were supported by Hebei Medical Science Research Key Project, No: 20221236. "

Please state what role the funders took in the study. If the funders had no role, please state: "The funders had no role in study design, data collection and analysis, decision to publish, or preparation of the manuscript."

If this statement is not correct you must amend it as needed.

Please include this amended Role of Funder statement in your cover letter; we will change the online submission form on your behalf.

4. In this instance it seems there may be acceptable restrictions in place that prevent the public sharing of your minimal data. However, in line with our goal of ensuring long-term data availability to all interested researchers, PLOS’ Data Policy states that authors cannot be the sole named individuals responsible for ensuring data access (http://journals.plos.org/plosone/s/data-availability#loc-acceptable-data-sharing-methods).

Data requests to a non-author institutional point of contact, such as a data access or ethics committee, helps guarantee long term stability and availability of data. Providing interested researchers with a durable point of contact ensures data will be accessible even if an author changes email addresses, institutions, or becomes unavailable to answer requests.

Before we proceed with your manuscript, please also provide non-author contact information (phone/email/hyperlink) for a data access committee, ethics committee, or other institutional body to which data requests may be sent. If no institutional body is available to respond to requests for your minimal data, please consider if there any institutional representatives who did not collaborate in the study, and are not listed as authors on the manuscript, who would be able to hold the data and respond to external requests for data access? If so, please provide their contact information (i.e., email address). Please also provide details on how you will ensure persistent or long-term data storage and availability.

[Note: HTML markup is below. Please do not edit.]

Reviewers' comments:

Reviewer's Responses to Questions

Comments to the Author

1. Is the manuscript technically sound, and do the data support the conclusions?

The manuscript must describe a technically sound piece of scientific research with data that supports the conclusions. Experiments must have been conducted rigorously, with appropriate controls, replication, and sample sizes. The conclusions must be drawn appropriately based on the data presented.

Reviewer #1: Yes

Reviewer #2: Yes

2. Has the statistical analysis been performed appropriately and rigorously?

Reviewer #1: Yes

Reviewer #2: Yes

3. Have the authors made all data underlying the findings in their manuscript fully available?

The PLOS Data policy requires authors to make all data underlying the findings described in their manuscript fully available without restriction, with rare exception (please refer to the Data Availability Statement in the manuscript PDF file). The data should be provided as part of the manuscript or its supporting information, or deposited to a public repository. For example, in addition to summary statistics, the data points behind means, medians and variance measures should be available. If there are restrictions on publicly sharing data—e.g. participant privacy or use of data from a third party—those must be specified.

Reviewer #1: Yes

Reviewer #2: Yes

4. Is the manuscript presented in an intelligible fashion and written in standard English?

PLOS ONE does not copyedit accepted manuscripts, so the language in submitted articles must be clear, correct, and unambiguous. Any typographical or grammatical errors should be corrected at revision, so please note any specific errors here.

Reviewer #1: No

Reviewer #2: Yes

5. Review Comments to the Author

Please use the space provided to explain your answers to the questions above. You may also include additional comments for the author, including concerns about dual publication, research ethics, or publication ethics. (Please upload your review as an attachment if it exceeds 20,000 characters)

Reviewer #1: 1.English expressions need to be edited more careful and more native, in this manuscript, there are some mistakes.

*Response:Thank you for your* *comments, we have made changes to the English expression.*

2. I suggest the authors should add a flowchart in the manuscript to show the process very well.

*Response:We added the flowchart as Figure 1.*

3. I suggest the authors should elaborate their motivation in the discussion section, as they use several common bioinformatics analysis methods and online tools. What is the novelty and technicality of their work?

*Response:Thank you for your comments,We revised the discussion section. The innovation of the paper is that we used machine learning to screen the core genes.*

3. Advancements in computational capabilities have led to the widespread application of machine learning techniques, especially deep learning, across various bioinformatics fields. Important computational models in these fields should be cited. Some recommended studies are helpful (PMIDs: 36305458, 34232474, 35817399, 36584603, 37525507, 37660567, 38040204, and 37466194).

*Response:Thank you for your suggestions. I have learned a lot from reading these articles carefully and quoted some of them (PMIDs: 36305458, 34232474, 36584603). The rest of the articles are very good, but considering the volume of the articles, I am very sorry that I could not quote them. Then the citation article must be preferred.*

5. The authors should carefully check and unify the information of references. Some references lack the information of volume or contain the wrong page number.

*Response:We have modified the reference format with errors, thank you for your comments.*

6. Methods section is relatively simple because of no detail about the analysis. The analysis methods and statistical parameters must be clearly emphasized (i.e, reasons for selecting the algorithms used, threshold values used in statistical analysis, etc.).

*Response:Thanks for your suggestion, we have added the details of the methods section.*

7. The authors claim that HRSP12 and DCXR may be potential biological markers and renal protective factors. I think more validation should be adopted. Especially, I want to see some associations can be confirmed by other method or biological experiments.

*Response:Thanks for the reviewer’s comments.We added qrt-PCR, cell-based experiments to knock down related genes, and CCK8 experimental validation. The results suggest that HRSP12 and DCXR may be potential biomarkers and renal protective factors.*

8. Future work and limitations of the proposed algorithm should be addressed detailly in the manuscript for further research.

*Response:Thanks for the reviewer’s comments.We explained this in detail in the discussion section*

Reviewer #2: This study investigates the relationship between cuproptosis genes and the immune microenvironment in diabetic nephropathy (DN). RNA sequencing data from DN and normal renal tissues were analyzed for differential gene expression and immune cell subtype infiltration. NLRP3 and CDKN2A were positively correlated with immune scores, while NFE2L2 and other genes were negatively correlated. Consensus clustering based on cuproptosis genes identified two clusters: C1 (low cuproptosis gene expression, high immune cell infiltration) and C2 (opposite characteristics). Key genes DCXR and HRSP12 were identified and used to construct a nomogram with high diagnostic accuracy for DN. These genes may serve as potential biomarkers and renal protective factors. There are some issues that need to be addressed before the article can be accepted.

1. What do “NLRP3, CDKN2A, NFE2L2, LIAS, LIPT1, DLD, DBT and DLST” represent in the Abstract section? What are their full names? When first mentioned, the author should provide their full names to help readers better understand.

*Response:Thank you for your comments. NLRP3, CDKN2A, NFE2L2, LIAS, LIPT1, DLD, DBT and DLST are the genes we screened, and their full names have been added in the abstract.*

2. The authors should add a flowchart in the manuscript to show the process very well.

*Response:We added the flowchart as Figure 1.*

3. The Methods section in the text lacks corresponding literature references. The author did not provide sources and citations for the Methods mentioned.

*Response:Thanks for your suggestion, we have cited the method sources and added related literature.*

4.The labeling of Figures in the paper is quite small. The contents within the figures are unclear. The author needs to carefully revise and modify the figure.

*Response:Thank you for your comments. We have made changes to the figures.*

5. The authors should carefully check and unify the information of references. Some references lack the information of page number, such as ref [26].

*Response:Thank you for your comments.We have modified the reference format with errors.*

6. The critique of current research limitations is somewhat lacking. The authors are encouraged to frame these deficiencies as directions for future research. Studies on gene/protein signaling networks using ODE-based theoretical modeling have played a crucial role in disease prediction (https://doi.org/10.1016/j.chaos.2023.114328, https://doi.org/10.1103/PhysRevE.108.064412, PMIDs: 33389663, 35958114). It would be beneficial if the authors could integrate and reference these four studies in this paper.

*Response:Thanks for your suggestions, we have benefited a lot from reading these studies. Based on the four studies, we have supplemented the limitations of the current studies in the discussion section.*

7.Besides, deep learning based algorithms offer valuable insights into diseases. It is essential to discuss and cite key computational models in these areas. Including some recommended studies would be beneficial (PMID: 38737196, doi:10.1016/j.swevo.2024.101567).

*Response:Thanks for your suggestions, we have cited these studies to enrich the content of the article.*

6. PLOS authors have the option to publish the peer review history of their article (what does this mean?). If published, this will include your full peer review and any attached files.

If you choose “no”, your identity will remain anonymous but your review may still be made public.

Do you want your identity to be public for this peer review? For information about this choice, including consent withdrawal, please see our Privacy Policy.

Reviewer #1: No

Reviewer #2: No

[NOTE: If reviewer comments were submitted as an attachment file, they will be attached to this email and accessible via the submission site. Please log into your account, locate the manuscript record, and check for the action link "View Attachments". If this link does not appear, there are no attachment files.]

While revising your submission, please upload your figure files to the Preflight Analysis and Conversion Engine (PACE) digital diagnostic tool, https://pacev2.apexcovantage.com/. PACE helps ensure that figures meet PLOS requirements. To use PACE, you must first register as a user. Registration is free. Then, login and navigate to the UPLOAD tab, where you will find detailed instructions on how to use the tool. If you encounter any issues or have any questions when using PACE, please email PLOS at <a href="mailto:figures@plos.org">figures@plos.org. Please note that Supporting Information files do not need this step.
