# Supplementary material for: Cuproptosis-related gene signatures define the immune microenvironment in diabetic nephropathy
Source: PLoS One. 2025 Jun 3;20(6):e0321636. doi: 10.1371/journal.pone.0321636 (PMC12133194; doi:10.1371/journal.pone.0321636)
Supplement: Attachment — Submitted filename: Responses to reviewer comments.docx [file pone.0321636.s010.docx]

Dear Editor

Thanks for your and the reviewers’ suggestions and comments concerning our manuscript. Those comments are all very valuable and helpful for improving our paper. We have revised the manuscript point by point according to your and the reviewers’ comments, and believe that the paper has now been improved greatly with your help. The changes are highlighted in red in the revised manuscript. Detailed responses to these comments are listed below this letter.

We hope our revisions are now satisfactory for acceptance. If needed, we are very willing to make further revision. We look forward to hearing from you soon.

With best wishes,

Yours sincerely,

Fan Lu

Department of nephrology,

The Fourth Hospital of Hebei Medical University

**Reviewer comments:**

**Reviewer #1 (Remarks to the Author):**

**previous comment：**

3.I suggest the authors should elaborate their motivation in the discussion section, as they use several common bioinformatics analysis methods and online tools. What is the novelty and technicality of their work?

Response:Thanks for the reviewer's suggestions. Many commonly used tools and analytical methods were indeed used in this study. From an innovative point of view, we discussed the relationship between Cuproptosis and DN for the first time, and functional experiments verified the protective effect of DXCR on kidney. We added a description in the discussion section of the article(page 17,lines 343 to 352).

*“Cuproptosis is related to the occurrence and development of diseases. The main pathological mechanism of DN is metabolic disorder and renal fibrosis induced by immune response. This study is the first to link Cuproptosis gene with DN, and explore the possibility of Cuproptosis affecting the pathogenesis of DN. The relationship between Cuproptosis gene and immune microenvironment in DN was revealed, and the heterogeneity of disease was explored. Two key genes, HRSP12 and DCXR, were not confirmed in previous studies to have a protective effect of DCXR on kidney. This study verified this conclusion from the results of cell experiments.”*

4.Advancements in computational capabilities have led to the widespread application of machine learning techniques, especially deep learning, across various bioinformatics fields. Important computational models in these fields should be cited. Some recommended studies are helpful (PMIDs: 36305458, 34232474, 35817399, 36584603, 37525507, 37660567, 38040204, and 37466194).

Response:Thanks to the suggestions of reviewers, these studies are very helpful to supplement the content of the article, and we have quoted all of them(page 20,lines 417 to 425 and page 22,lines 454 to 462).

*“Unprecedented development of bioinformatics and machine learning algorithms, machine learning technology, especially deep learning, is widely used in various bioinformatics fields, and researchers in this field have proposed many important computational models, for example, GCNCRF algorithm predicts human lncRNA-miRNA interaction, Network distance analysis model lncRNA-miRNA association prediction (NDALMA) and deep learning prediction model DMFGAM were used to predict Human ether A-Go-Go-related gene (hERG) blockers, providing a new method for screening disease biomarkers[40-42]. ......The study of gene/protein signaling networks using ODE-based theoretical models has played a crucial role in disease prediction[49-52]. There are also some computational models based on deep learning that have high value for the in-depth study of diseases[53,54]. For example, a non-negative matrix decomposition deep learning model named MDA-AENMF is used to predict potential links between metabolites and disease[55], and researchers have proposed a new RNA-SEQ analysis framework called scAAGA that adaptively learns important genetic signatures from RNA-SEQ data to improve the clustering effect[56-59].”*

6.Methods section is relatively simple because of no detail about the analysis. The analysis methods and statistical parameters must be clearly emphasized (i.e, reasons for selecting the algorithms used, threshold values used in statistical analysis, etc.).

Response:Thanks to your suggestions, we have added some method details and restated some methods(page 8).

*“The cut height was set to 0.25 and each module contained at least 30 genes. ......*

*Construction of predictive model based on multiple machine learning methods*

*Based on two different cuproptosis genes clusters, we applied the “caret”R packages (version 6.0.91) for establishing machine learning models including random forest model (RF), support vector machine model (SVM), generalized linear model (GLM), and eXtreme Gradient Boosting (XGB)[25].The distinct clusters were considered as the response variable, and the cluster-specific DEGs were selected as explanatory variables. The 83 DN samples were randomly classified into a training set (60%, N=50) and a validation set (40%, N=33). The caret package automatically tuned the parameters in these models by grid search, and all of these machine learning models were performed with default parameters and assessed via 5-fold cross validation. The “DALEX” package (version 2.4.0) was carried out to interpret the aforementioned four machine learning models and visualize the residual distribution and feature importance among these machine learning models. The “pROC” R package (version 1.18.0) was performed to visualize the area under ROC curves. Consequently, the optimal machine learning model was determined and the top two important variables were considered as the key predictive genes associated with DN. Finally, The ROC curves analysis were performed to verify the diagnostic value of the diagnostic model.”*

8. Future work and limitations of the proposed algorithm should be addressed detailly in the manuscript for further research.

Response:Thanks for your suggestion, we have added the shortcomings of our own research in the discussion section.(page 23,lines 463 to 469)

*“Compared with previous studies, our study has some shortcomings. First, our current study is based on a comprehensive bioinformatics analysis and cytological trials that require additional clinical or experimental evaluation of DN tissues to validate DCXR and HRSP12 expression levels. In addition, more detailed clinical data is needed to confirm the performance of predictive models. Finally, more detailed experiments are needed to elucidate the underlying mechanisms of DCXR and HRSP12 with DN.”*

1. The authors didn't know how to write response letter. I always need to search for the revised content in the manuscript. You should list all the corresponding revisions in the letter.

Response:Sorry for the trouble caused to your work, I have shown the problems that need to be shown and modified on the above again.

2. The authors even write two “3” in the response to Reviewer #1.

Response:Thank you for your suggestion. This is due to an inspection error. We have carefully checked this response letter.

3. The authors fail to elaborate their motivation in the discussion section. There are many papers on using machine learning to screen the core genes, so there is not as main innovation of the paper.

Response:Thank you for your suggestion. We explained in the discussion section that this study is innovative. Although common tools and analytical methods are used, this is the first study on the association between Cuproptosis and DN disease(page 17,lines 343 to 352).

*“Cuproptosis is related to the occurrence and development of diseases. The main pathological mechanism of DN is metabolic disorder and renal fibrosis induced by immune response. This study is the first to link Cuproptosis gene with DN, and explore the possibility of Cuproptosis affecting the pathogenesis of DN. The relationship between Cuproptosis gene and immune microenvironment in DN was revealed, and the heterogeneity of disease was explored. Two key genes, HRSP12 and DCXR, were not confirmed in previous studies to have a protective effect of DCXR on kidney. This study verified this conclusion from the results of cell experiments.”*

4. The authors should revise carefully corresponding to my previous comment #4. All the related paper should be cited.

Response:Thanks for your suggestion, we have revised and quoted all relevant papers(page 20,lines 417 to 425 and page 22,lines 454 to 462).

*“Unprecedented development of bioinformatics and machine learning algorithms, machine learning technology, especially deep learning, is widely used in various bioinformatics fields, and researchers in this field have proposed many important computational models, for example, GCNCRF algorithm predicts human lncRNA-miRNA interaction, Network distance analysis model lncRNA-miRNA association prediction (NDALMA) and deep learning prediction model DMFGAM were used to predict Human ether A-Go-Go-related gene (hERG) blockers, providing a new method for screening disease biomarkers[40-42]. ......The study of gene/protein signaling networks using ODE-based theoretical models has played a crucial role in disease prediction[49-52]. There are also some computational models based on deep learning that have high value for the in-depth study of diseases[53,54]. For example, a non-negative matrix decomposition deep learning model named MDA-AENMF is used to predict potential links between metabolites and disease[55], and researchers have proposed a new RNA-SEQ analysis framework called scAAGA that adaptively learns important genetic signatures from RNA-SEQ data to improve the clustering effect[56-59].”*

5. The flowchart in Fig.1 is too simple to show the process of data collection and method implementation very well, please improve it.

Response:Thanks for your suggestion, we have reworked the flow chart.

6. Why the authors mention that HRSP12 and DCXR may be potential biomarkers and renal protective factors to my previous comment #7. If the authors can't get positive results, then what's the point of the experiment.

Response:From the qrt-PCR and CCK8 proliferation experiments, we found that HRSP12 and DCXR were highly expressed in the DN model, and the two genes played a role in inhibiting cell proliferation in the DN model (*P*<0.05), and we concluded that HRSP12 and DCXR were potential biomarkers and kidney protective factors.

7. There are still some grammatical errors in the article, and the expression of some of the content is not clear enough. The authors need to check the manuscript carefully and make corresponding revision.

Response:Thanks for your suggestions, we have revised the grammar of the article to improve the corresponding expression.

8. Why there are two new co-first authors in the revised manuscript?

Response:The work of two new authors is more prominent in the calculation and experimental part. The original first author did not participate in the follow-up research, and the team decided to modify the author list after unanimous discussion.
